# Bond Strength Assessment of Normal Strength Concrete–Ultra-High-Performance Fiber Reinforced Concrete Using Repeated Drop-Weight Impact Test: Experimental and Machine Learning Technique

**DOI:** 10.3390/ma17123032

**Published:** 2024-06-20

**Authors:** Sadi I. Haruna, Yasser E. Ibrahim, Ibrahim Hayatu Hassan, Ali Al-shawafi, Han Zhu

**Affiliations:** 1 Engineering Management Department, College of Engineering, Prince Sultan University, Riyadh 11586, Saudi Arabia; ymansour@psu.edu.sa (Y.E.I.); hanzhu2000@tju.edu.cn (H.Z.); 2 Institute of Agricultural Research, Ahmadu Bello University, Zaria 810006, Nigeria; ihhassan@abu.edu.ng; 3 School of Civil Engineering, Tianjin University, Tianjin 300350, China

**Keywords:** normal strength concrete, UHPFRC, impact strength, surface treatment, machine learning algorithms

## Abstract

Ultra-high-performance concrete (UHPC) has been used in building joints due to its increased strength, crack resistance, and durability, serving as a repair material. However, efficient repair depends on whether the interfacial substrate can provide adequate bond strength under various loading scenarios. The objective of this study is to investigate the bonding behavior of composite U-shaped normal strength concrete–ultra-high-performance fiber reinforced concrete (NSC-UHPFRC) specimens using multiple drop-weight impact testing techniques. The composite interface was treated using grooving (Gst), natural fracture (Nst), and smoothing (Sst) techniques. Ensemble machine learning (ML) algorithms comprising XGBoost and CatBoost, support vector machine (SVM), and generalized linear machine (GLM) were employed to train and test the simulation dataset to forecast the impact failure strength (*N*2) composite U-shaped NSC-UHPFRC specimen. The results indicate that the reference NSC samples had the highest impact strength and surface treatment played a substantial role in ensuring the adequate bond strength of NSC-UHPFRC. NSC-UHPFRC-Nst can provide sufficient bond strength at the interface, resulting in a monolithic structure that can resist repeated drop-weight impact loads. NSC-UHPFRC-Sst and NSC-UHPFRC-Gst exhibit significant reductions in impact strength properties. The ensemble ML correctly predicts the failure strength of the NSC-UHPFRC composite. The XGBoost ensemble model gave coefficient of determination (*R*^2^) values of approximately 0.99 and 0.9643 at the training and testing stages. The highest predictions were obtained using the GLM model, with an *R*^2^ value of 0.9805 at the testing stage.

## 1. Introduction

Because of its superior compressive strength and durability, UHPC is becoming increasingly popular in many building construction industries. These ideal UHPC characteristics can decrease the size and self-weight of a structure remarkably. UHPC is frequently used as a joint to combine concrete members [1,2] and a repair and retrofit material for damaged concrete structures [1]. UHPC has been used to enhance and strengthen concrete elements, including bridges and decks; this increases the behavior of the existing reinforced concrete structures, including hardened and durability properties. The NSC-UHPC composites involve the interface between the substrate and new cementitious materials with weak areas [1,2]. The bonding behavior at the interface considerably affects the overall performance of the concrete structure [3]. The interface of NSC-UHPC composites is under a self-equilibrated state of tension collective with shear [4].

The normal stress states at the interface, such as tension, shear, and shear–compression combinations, are essential. Therefore, many studies have investigated and modeled the bond behavior between old and new cementitious materials using different laboratory methods, including (i) tension tests such as pull-off, direct tension, and splitting, and (ii) shear tests that include direct shear and slant shear. The experiments described above were performed under static loading conditions. The interfacial bond of composite NSC-UHPC is primarily examined under static load application. However, UHPC material is widely used to repair concrete structures such as military structures, bridge columns, and road and runway facilities that are usually exposed to impact loads. Studying the bond performance of NSC-UHPC composite under impact loads can aid in selecting the best material for NC structural repair and retrofitting application.

Yu et al. [5] investigated the debonding failure pattern of the NSC-UHPC composite using a multi-scale technique. The results revealed that the NC substrate’s interface roughness and strength increased mechanical performance. Yang et al. [6] examined the bending behavior of a precast NSC-UHPC specimen. The results showed that the hybrid NSC-UHPC exhibits sufficient capacity, stiffness, etc., and the composite section displayed large fracture widths. Jongvivatsakul et al. [7] reported improved bond strength between carbon-fiber-reinforced polymer plates and concrete using carbon nanotube-reinforced epoxy composites. The U-shaped drop-weight impact testing approach is a newly developed impact testing technique used to evaluate the impact properties of cementitious materials [8,9,10]. The approach evaluated impact strength using a unique U-shaped sample and a repeated drop weight. In addition, the types and impact properties of UHPC under diverse loading circumstances were examined in past studies, including low-velocity impact [11], projectile loads [12,13], dynamic splitting [14], dynamic mechanical method [15], and multiple impact loads [16]. Yu et al. [17] used pendulum loads to determine the impact strength of UHPFRC samples. They discovered that the fiber length is the primary cause of the increased energy dissipation capacity in the UHPC.

The traditional approaches for forecasting these features are based on empirical interactions established from broad lab research. Conversely, the complication and non-linear parameters determining the impact resistance of concrete require advanced prediction techniques. The capability of ML revealed the remarkable capability to handle complicated relationships in a broad database, which demonstrates a pronounced perspective in several engineering fields. Almustafa and Nehdi [18] developed an ensemble tree-based model for estimating the structural behavior of reinforced concrete (RC) columns under blast loads. The authors predicted the blast characteristics of individual elements of RC structures to eventually construct an adaptable model for the global performance of the complete RC structures. Zhang et al. [13] predicted the impact and blast strength of UHPC using the Karagozian and Case (K&C) material model. The technique’s prediction ability was demonstrated by apprehending numerous experimental datasets. Cao et al. [19] forecasted the impact strength of concrete containing fibers using the adaptive neuro-fuzzy inference system (ANFIS) model. The UHPC’s dynamic mechanical characteristics were assessed using the ML model [20]. Shao et al. [21] evaluated the UHPC’s penetrating resistance encapsulated in a ceramic ball exposed to projectile stress. However, little research has investigated the impact behavior of UHPFRC using repeated drop-weight impact tests and an artificial intelligence-based algorithm to analyze impact resistance. Therefore, the objective of this study is to assess the bonding strength of the NSC-UHPFRC composite under multiple drop-weight impact tests. The composite NSC-UHPFRC’s interface was treated using three surface treatment methods, which include normal fracture (Nst), smooth (Sst), and grooves surface (Gst). Moreover, two ensemble models, Catboost and XGboost, and classical models (SVR and GLM models) were used to predict the impact strength (*N*2) of the U-shaped NSC-UHPFRC composite. The study explored the suitability of studying the bond strength properties of composite materials under impact stress.

## 2. Materials and Methods

### 2.1. Materials

#### 2.1.1. NSC and UHPFRC

Table 1 presents the mixed proportion of the NSC and UHPFRC materials used in this study. Ordinary Portland cement (OPC) grade 52.5, confirmed with cement composition requirements specified in GB175-2007 [22]. The NSC mixture was prepared following Chinese standard JTG55-2011 [23]; the NSC mixture was designed to achieve a compressive strength of 40 MPa. The NSC constituent materials include natural river sand as fine aggregate and crushed stone as medium aggregate (10 mm particle size). At the same time, the UHPFRC mixture was prepared according to GB175-2007 [22], designed to achieve a compressive strength of 125 MPa. The constituent material of UHPC includes OPC, silica fumes (SF), slag, quartz powder was bought from Zhixiang Industrial Trading Co., Ltd. (Yongdeng, China), and a water-reducing agent was sourced from Jiangsu Subot New Materials Co., Ltd. (Nanjing, China),. The performance parameters of the steel fiber manufactured by Ganzhou Daye Metal Fiber Co., Ltd. (Ganzhou, China) was added to the UHPC mixture by volume fraction as summarized in Table 2. 

#### 2.1.2. Sample Fabrications 

**Figure 1:**
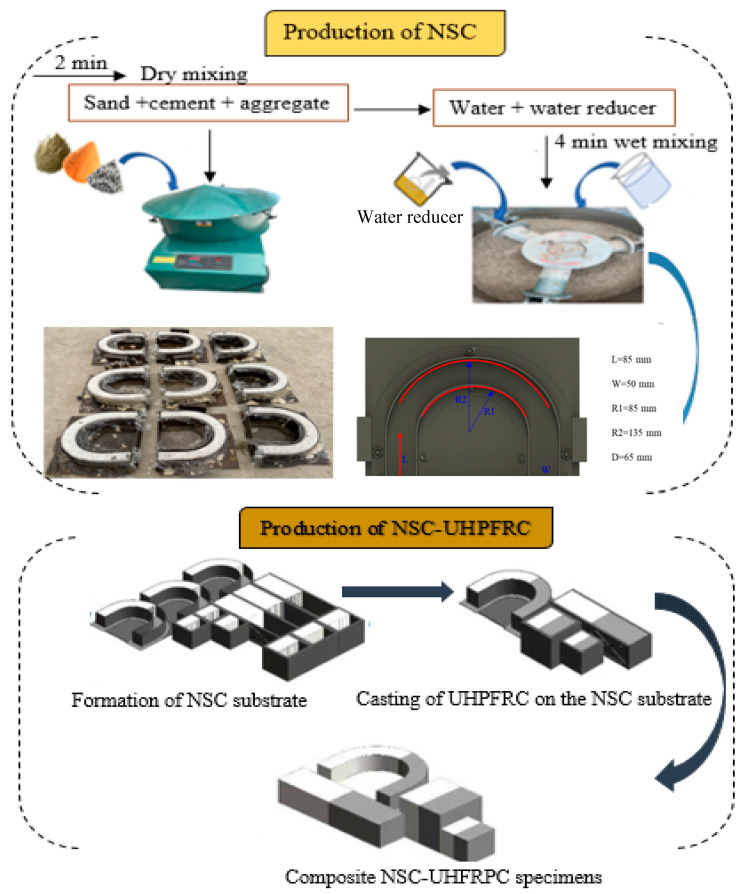
Schematic procedure for the production of NSC-UHPFRC specimens.

**Figure 2:**
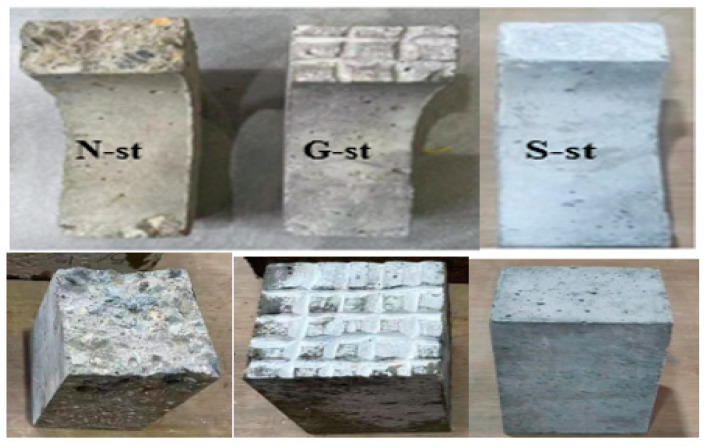
Surface treatment techniques adopted in this study.

This study fabricated U-shape cubes (100 × 100 × 100 mm^3^) and beam composite specimens (100 × 100 × 400 mm^3^) by bonding half the NSC substrate and half the UHPFRC layer together to form composite NSC-UHPFRC specimens. The preparation procedures for the production of the NSC-UHPFRC composite are illustrated in Figure 1. Three surface treatment methods were adopted at the interface based on the natural fracture, smooth, and grooves surfaces (see Figure 2). For each specimen condition, twenty (20) control and composite NSC-UHPFRC specimens were fabricated and tested for multiple drop-weight impacts to assess the bonding behavior at the interface. The specimens treated with different surface interfaces were produced after casting the NSC U-shaped specimen in the mold and cured for 28 d. The cured samples, including the cube and beams, were then cut into two pieces considering smoothed, natural fracture, and grooved surfaces. The NSC substrate surface was treated using longitudinal grooving and smoothing techniques. The last group was made by just breaking the NSC samples naturally. The half pieces for each surface treatment were then put back into the mold to cast the remaining half of the UHPFRC. After casting the composite NSC-UHPFRC, the samples were then kept at a standard curing room temperature for another 28 d for the UPHFRC part to attain its maximum before testing.

### 2.2. Testing Methods

Previous studies [24] have indicated many testing methods for investigating the bond behavior at the interface of composite materials. These are classified into two: (I) testing under the synergy effect of shear and compression stress, for instance, the most reliable bond strength result may not be determined using the slant shear test due to the existence of compression force. (II) Testing under pure sheer stress includes splitting tensile, 3-point flexural test, direct shear, etc. This research attempted to evaluate the bond behavior of the NSC-UHPFRC composite at the interface under multiple drop-weight impact testing techniques. 

#### Multiple Drop-Weight Impact Tests

**Figure 3:**
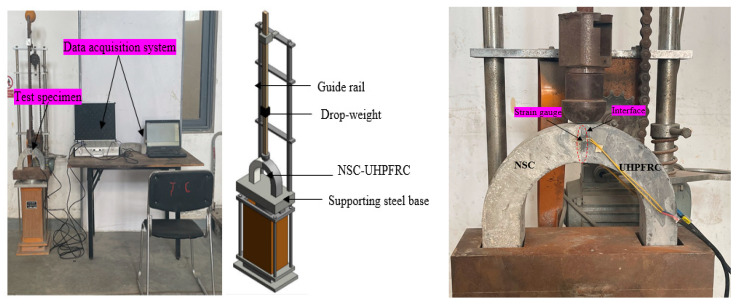
Multiple drop-weight Impact tests.

Figure 3 presents the experimental process for impact tests. The NSC-UHPFRC specimens were exposed to repeated impact loads using a hammer weighing 2.1 kg, which was released from a 457 mm distance. The impact test was carried out following a modified version of the ACI 544- test approach [25]. The impact loads that induced the first crack and failure strength were recorded as *N*1 and *N*2, respectively, reflecting the bond resistance subjected to dynamic load. The absorbed impact energy is determined using Equations (1) and (2) for impact mass (*w*), acceleration due to gravity (*g*), falling mass velocity (*v*), from height (*h*) at the first and complete failure stages, respectively. The test specimens are instrumented with strain gauges to enable the detection of the occurrence of the first crack, which is transmitted to the data acquisition system.

(1) $$IE_{1}=N_{1}.wgh=N_{1}\frac{wv^{2}}{2}$$

(2) $$IE_{2}=N_{2}.wgh=N_{2}\frac{wv^{2}}{2}$$

### 2.3. Machine Learning Algorithms

In this paper, two ensemble machine learning models (XGboost and CatBoost), SVM and GLM, were used to forecast *N*2 at the fracture stage of the test specimen under multiple drop-weight impact tests python version 3.10 The developed models were used to train the datasets acquired from this study and previous literature [26]. To model *N*2, sensitivity analysis was used to select the input variables in the XGBoost, CatBoost, SVR, and GLM models, as depicted in Equation (3)

(3) $$failure\;strength\;\left(N_{2}\;\left(blows\right)\right)=\left\{\begin{array}{c} XGBoost=f(Cfc+P+\rho +N_{1})\\ CatBoost=f\left(Cfc+P+\rho +N_{1}\right)\\ SVR=f\left(Cfc+P+\rho +N_{1}\right)\\ GLM=f\left(Cfc+P+\rho +N_{1}\right) \end{array}\right\}$$

where *f* defines the function of the input variable, *Cfc* is the compressive strength of the composite, *P* = flexural load, *ρ* is the density, and *N*1 is the first crack strength.

The modeling dataset was divided into a ratio of 70:30 for the training and testing stages. The efficiency of the two models was validated using a 10-fold cross-validation approach. This approach is reported in the literature [27] as the best method for providing unbiased model evaluation for a small dataset. The dataset was also normalized using Equation (4) to enhance its integrity and decrease redundancy.

(4) $$x_{n}=\frac{x-x_{min}}{x_{max}-x_{min}}$$

where *x_n_* is the scaled value, *x* is the un-normalized value, *x_min_* is the smallest value, and *x_max_* defines the largest value in the dataset.

#### 2.3.1. CatBoost

CatBoost, referred to as categorical boosting, is an enhanced ML technique based on the gradient-boosting tree structure. It is built on the symmetric decision tree technique and largely handles the issues of efficiently managing categorical information, gradient bias, and estimate offsets. CatBoost tries to improve the algorithm’s skills and general capabilities by combining the results of all trees to get the final solution [28]. CatBoost generates plus one independent random sample permutations from a given training set. The main objective of the CatBoost implementation was to tackle prediction shift issues. Manual settings were considered when choosing the hyperparameters for the experiment. The iterations were set to 50,000, the maximum depth to 3, the learning rate to 0.1, the loss function to “RMSE, MSE, MAE, and *R*^2^”, and other default settings were maintained at default values.

#### 2.3.2. XGBoost

XGBoost is a robust supervised tree-based ML model widely employed in several disciplines because of its high accuracy and ability to overcome overfitting difficulties commonly encountered in other tree-based approaches [29,30]. XGBoost was built using the Gradient Boosting technique, which improves performance by mixing weak models. This technique processes the input parameters and then builds a model to implement the following tasks: ranking, regression, and classification. Here, we engaged the XGBoost to handle regressive prediction tasks. A compressed mathematical design of the loss function using the XGBoost hyperparameters is depicted in Equation (5).

(5) $$L=\sum_{j=1}^{T_{m}}\left[G_{jm}W_{jm}+\frac{1}{2}\left(H_{jm}+\lambda_{R}\right){W^{2}}_{jm}+\alpha_{R}\left|W_{jm}\right|\right]+\gamma T_{m}$$

where L is the loss function, $\alpha_{R}$ and $\lambda_{R}$ indicate *L*1 and *L*2 regularization factors, respectively, and γ designates the penalization variable. $H_{jm}$ and $G_{jm}$ signify the sum of hessian and the sum of gradient, respectively, for the optimum weight for each region, *j* ($H_{jm}$ was applied to obtain the smallest sum of child weights) and $W_{jm}$ represents the optimum weights. $T_{m}$ denotes leave tree over a maximum depth of tree $D_{max}$. 

#### 2.3.3. Support Vector Regression (SVR)

**Figure 4:**
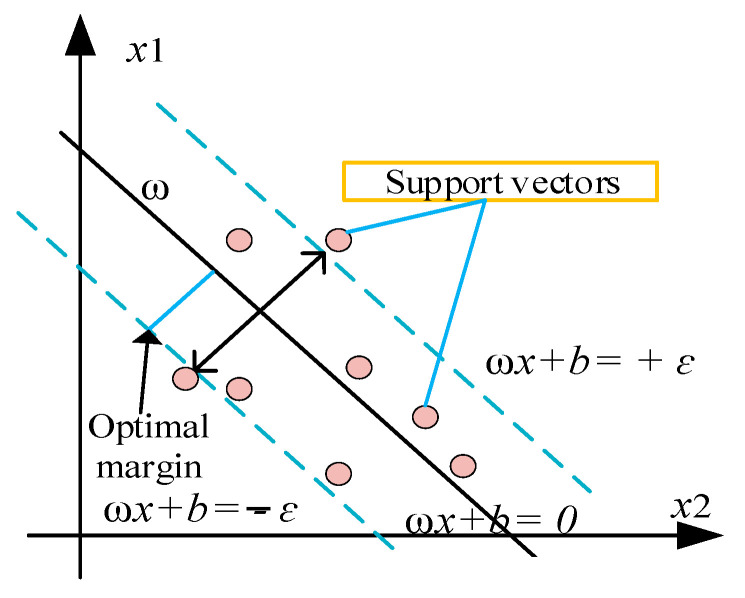
Structure of SVM.

SVR is an adaptation of the SVM and has been successfully applied to regression evaluation in a number of scientific and technical applications. The primary goal of an SVR model is to accurately estimate the output parameter, $\left\{p_{i}\right\}$, in accordance with a set of input parameters, $\left\{y_{i}\right\}$, by fitting a regression function, P=f(y), correctly. The training dataset is represented by the expression$\;M={(y}_{1},\;p_{1}),\;{(y}_{2},\;p_{2}),\ldots ,\;(y_{k},\;p_{k})$, where $p_{i}$ ϵ $R_{K}$ denotes the desired quantity and $y_{i}$ ϵ $R_{K}$ is a vector representing the input features. For non-linearly connected data to the preferred output variables, as is the case in nonlinear scenarios observed in the real world, a nonlinear mapping function $x_{i}(y)$ as given in Equation (6) [31] can be used to build the linear relation in the high-dimensional feature space. The structure of the SVR model is shown in Figure 4.

(6) $$f\left(y\right)=\sum_{i=1}^{n}w_{i}x_{i}\left(y\right)+b$$

where b is the bias, $w_{i}x_{i}\left(y\right)$ is the function referred to as the feature, and the dot product in the feature space is F. It is based on the structural risk minimization theory [32].

#### 2.3.4. Generalized Linear Model (GLM)

The GLM is frequently applied to binary or count data modeling [33]. GLM can be considered a nonlinear regression model. We built a predictive model using GLM and a Gamma (Γ) distribution. GLM can be characterized as nonlinear due to the characteristics of the CRI distribution in the lexicon of used data. An explanation of the GLM formulation is as follows:

(7) $${\;y}_{i}=\sum_{k=1}^{K}\beta_{k}x_{ki}+\beta_{0}$$

where a linear function of the explanatory variable (input) $x_{ki}$ with input coefficients $\beta_{k}$ and bias value $\beta_{0}$ models the response $y_{i}$. The expectation of the dependent variable $\mu_{i}=E(y_{i})$ is operated upon by an invertible link function $g(\mu_{i}.)$ to a linear predictor $\left(y_{i}\right)$.

(8) $$g\left(\mu_{i}\right)=y_{i}=\sum_{k=1}^{K}\beta_{k}x_{ki}+\beta_{0}$$

#### 2.3.5. Performance Evaluation Measures

**Figure 5:**
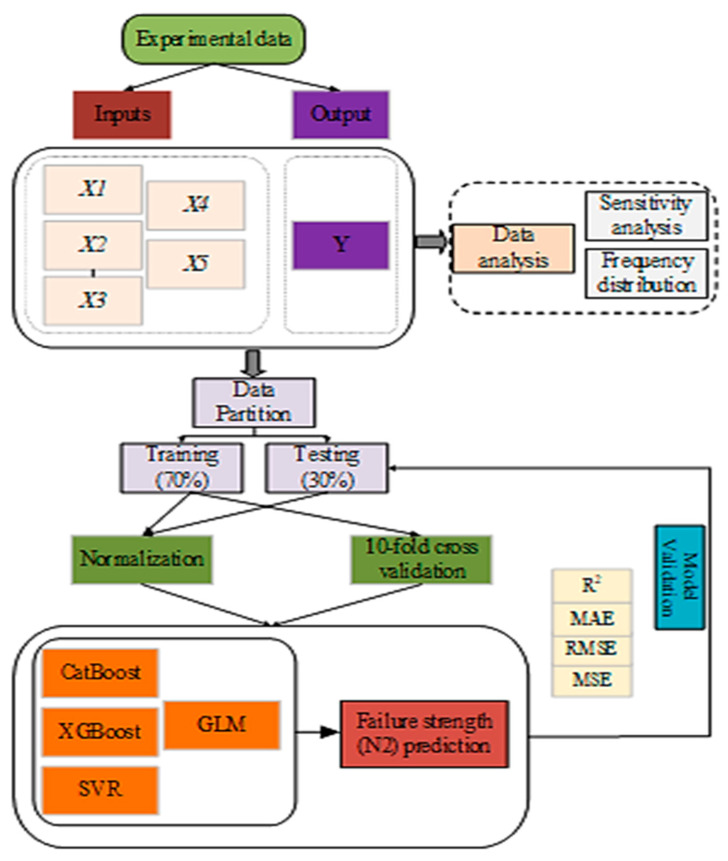
Flow chart of the model development.

To evaluate the efficiency of the ML models, four performance measures were used, including coefficient of determination (*R*^2^), mean absolute error (MAE), mean square error (MSE), and root mean square error (RMSE). The expression of the matrix is described by Equations (9) to (11). Therefore, the accuracy of the model noted in the indicators was used to determine which machine learning models were the best. The modeling process is depicted in Figure 5.

(9) $$R^{2}=\left[\frac{\sum_{i=1}^{n}\left(q_{i}-\bar{q}\right)\left(p_{i}-\bar{p}\right)}{\sum_{i=1}^{n}{\left(q_{i}-\bar{q}\right)}^{2\sum_{i=1}^{n}{\left(p_{i}-\bar{p}\right)}^{2}}}\right]$$

(10) $$MAE=\frac{\;1}{2}\sum_{i=1}^{n}\left|q_{i}-p_{i}\right|\;$$

(11) $$MSE=\frac{1}{n}\sum_{i=1}^{n}{\left(q_{i}-\bar{q_{i}}\right)}^{2}$$

## 3. Results and Discussions

### 3.1. Impact Resistance of NSC-UHPFRC 

**Figure 6:**
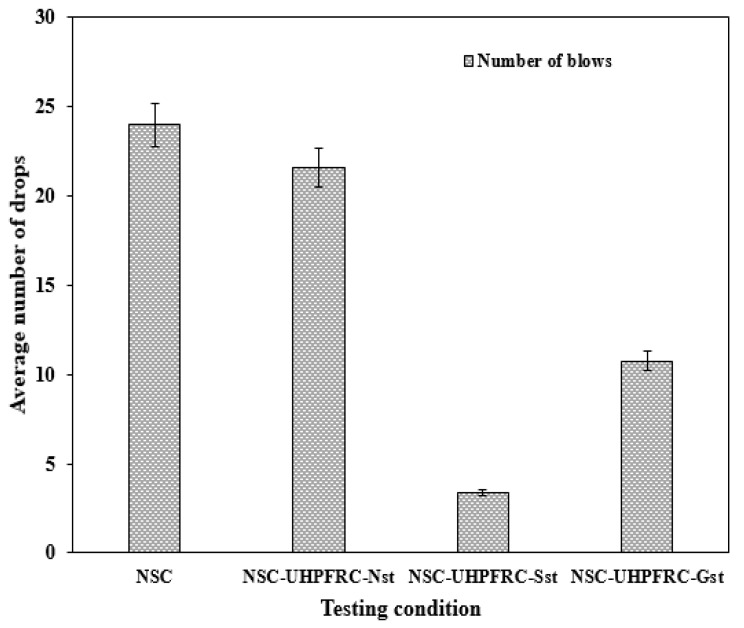
The impact strength (*N*1) of NSC-UHPFRC specimens.

**Figure 7:**
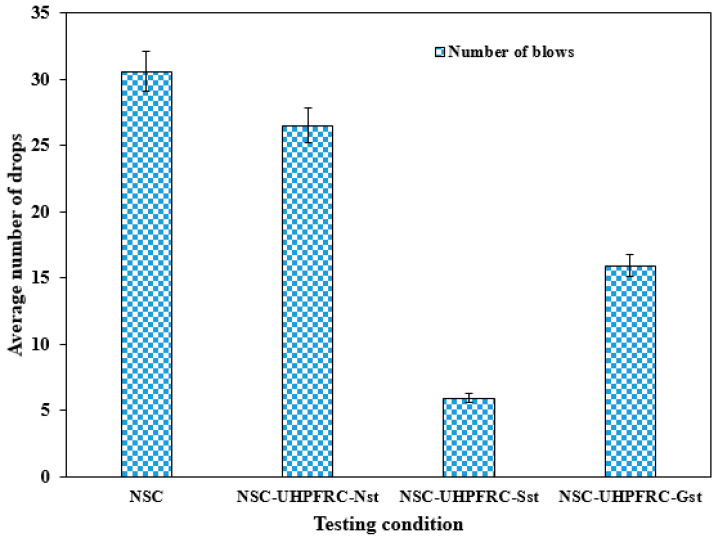
The impact strength (*N*2) of NSC-UHPFRC specimens.

Figure 6 and Figure 7 present the impact properties of NSC-UHPFRC composites under different testing conditions. The interface of composite NSC-UHPFRC specimens was treated with three different surface treatments. The effects of these surface treatments assess the composite bond’s ability to resist repeated impact loads. The number of drops that caused the first crack strength (*N*1), failure strength (*N*2), and equivalent absorbed impact energy for the four testing conditions are shown in Figure 6 and Figure 7, respectively. Equations (1) and (2) were applied to determine the absorbed impact energy as the two cracking stages were calculated based on drop weight and falling height. From Figure 7, it can be noted that the reference NSC specimen demonstrates a high drop number before the initial crack (*N*1) occurrence, with the average number of drops equal to 24 blows, followed by the specimen treated with natural fracture surface (NSC-UHPC-Nst). The U-shaped NSC-UHPFRC-Gst specimens reveal an average number of drops to initial the first crack strength of 11 blows, 118% lower than that of the control sample. The U-shaped composite formed with a smooth surface at the interface (NSC-UHPFRC-Sst) showed the lowest ability to resist the impact load at both the first and failure stages. The mean number of drops (*N*1) of the control NSC is 608.9% higher than that of the NSC-UHPFRC-Sst specimen. Similarly, the ability of the U-shaped composite to resist impact load caused complete failure of the specimens, which followed a similar pattern as the first crack strength. Reference NSC had the peak mean number of drops that caused complete failure, followed by NSC-UHPFRC-Nst, and then NSC-UHPFRC-Gst; the lowest average number of blows was recorded against NSC-UHPFRC-Sst specimens (see Figure 6). This finding shows that surface treatment plays a significant role in ensuring sufficient bond strength at the interface of the NSC-UHPFRC composite. The bond behavior between the NSC substrate under natural fracture and the UHPFRC layer can provide sufficient bond strength at the interface, resulting in a monolithic structure that can withstand dynamic loads under impact loads, as a slight decrease in the impact strength was obtained in the NSC-UHPFRC-Nst specimen compared to the reference NSC specimen. 

The ductility index of the test specimen is defined as the ratio of the post-crack strength (*N*2−N1) to the first-crack strength (*N*1), which explains the U-shaped NSC-UHPFRC specimen’s toughness after cracking [34,35]. The expression is given in Equation (9)

(12) $$Ductility\;index\;(\eta )=\frac{\left(N2-N1\right)}{N1}$$

**Figure 8:**
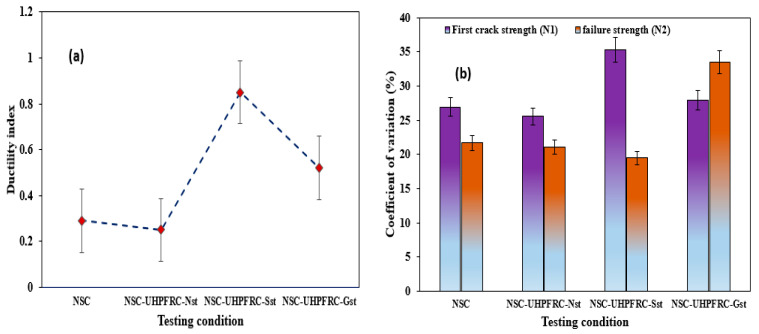
(**a**) Ductility index and (**b**) COV of the impact strength data.

Figure 8a presents the average ductility index (DI) value obtained under each testing condition, comprised of twenty (20) specimens demonstrating the ability of NSC-UHPFRC to absorb kinetic energy [34,36]. Because of the presence of SF in the UHPFRC, the ductility of the composite U-shaped was improved, which transformed the composite specimens to a more ductile state and enhanced the impact strength of the NSC-UHPFRC. The DI values of the NSC-UHPFRC for each testing condition are less than unity. The highest average DI value of 0.85 was obtained for NSC-UHPFRC-Sst specimens, attributed to the higher post-crack strength obtained in this group. The reference NSC, NSC-UHPFRC-Nst, and NSC-UHPFRC-Gst specimens had DI values of 0.29, 0.25, and 0.52, respectively. Figure 8b shows the COV of the impact strength data obtained at the two cracking phases (*N*1 and *N*2). As presented in Figure 8b, the NSC-UHPFRC-Sst specimen revealed the highest coefficient of variation of 35.29% at the initial crack point, with the lowest value of 19.51% at the failure stage (*N*2) among all testing groups. Interestingly, the maximum COV obtained in this work is lesser than the value achieved in many past studies [37,38] that utilized the drop-weight impact testing approach ACI 544-2R [25]. The reduction in COV is due to adopting a U-shape, which agrees with previous studies [8,39]. The overall impact test results are summarized in Table 3.

### 3.2. NSC

**Figure 9:**
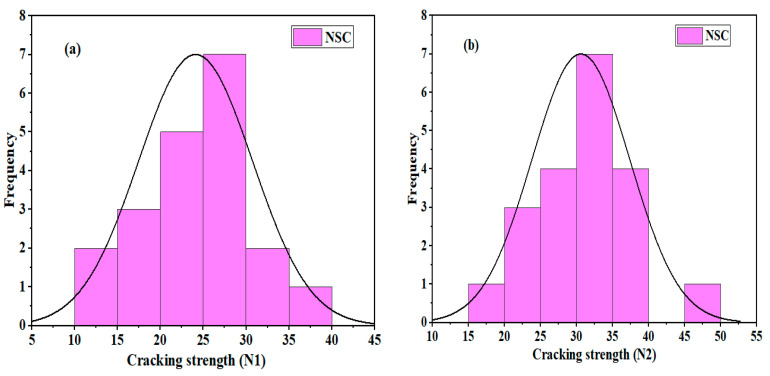
Distribution plots of impact test results for NSC: (**a**) *N*1 and (**b**) *N*2.

**Figure 10:**
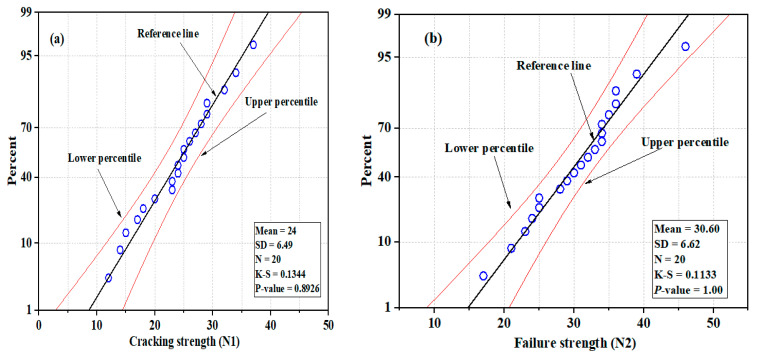
Normal probability plots of impact test results for NSC: (**a**) *N*1 (**b**) *N*2.

The control U-shaped NSC specimens showed maximum impact strength compared to the NSC-UHPFRC composite. The *N*1 values were in the range of 12 blows to 37 blows; the majority of test samples in this group demonstrated a great ability to resist impact stress prior to the appearance of the first crack. At the failure stage, the minimum and maximum blows were 17 and 46 blows, respectively. The statistical indicators, mean, SD, and COV were 24, 6.49 blows, and 26.92%, respectively. Figure 9 and Figure 10 present the distribution and probability plots of the impact strength data of the control NSC. The distribution graphs consisted of frequency histograms and superimposed normal curves. The test results at the two cracking stages nearly followed the normal distribution, as depicted in Figure 9a,b. The data were distributed near the mean value. The probability plots at the two cracking phases are shown in Figure 10a,b. It was observed that data points converged at the fitted line. The Kolmogorov–Smirnov (K-S) test was used to prove the normal distribution outcomes at a 0.05 significance level, and the results indicated that *N*1 had a *p*-value = 0.8926 and *N*2 had a *p*-value = 1. The results are consistent with normal distribution analysis.

### 3.3. NSC-UHPFRC-Nst

**Figure 11:**
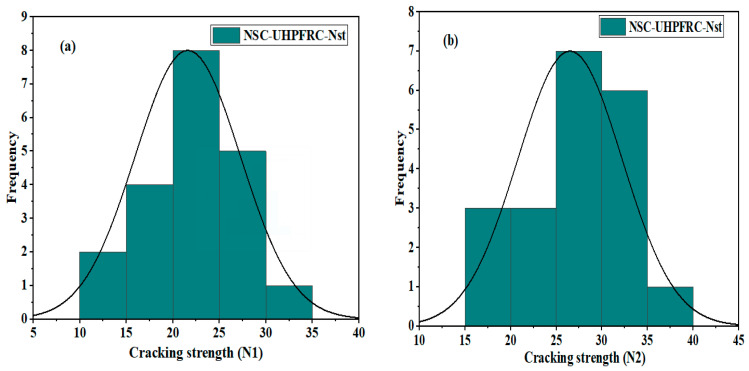
Distribution plots of NSC-UHPFRC-Nst impact test results: (**a**) *N*1 and (**b**) *N*2.

**Figure 12:**
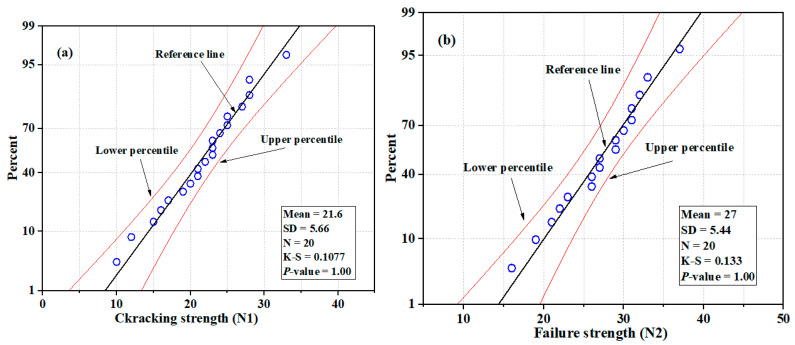
The probability plots of impact test results for NSC-UHPFRC-Nst: (**a**) *N*1 (**b**) *N*2.

The impact test results for U-shaped NSC-UHPFRC at the first and fracture stages were in the range of 10–28 blows for N1 and 15–37 blows for *N*2, as summarized in Table 4, with mean and SD values of 21.6 blow and 5.52 blows, respectively. For *N*1, the mean and standard deviation at the fracture stage stood at 26.5 blows and 5.58 blows, respectively. The impact test results of this group showed a slight reduction in the coefficient of variation compared to the control specimens; COV values of 25.54% and 21.06% were obtained for N1 and *N*2, respectively. Most of the test specimens in this group showed a high ability to resist impact loads that initiate the first crack during testing, as presented in Table 4. The normal distribution and probability plots of NSC-UHPFR for *N*1 and *N*2 are presented in Figure 11 and Figure 12. The plots show the impact strength data at the first and fracture stages follow a normal distribution, with distribution values located near the mean values in the overlaid curve, and the majority of data points concentrated along the fitted line, which is nearly normal. The K-S test result was at a 95% confidence level for *N*1 and *N*2, and the *p*-values were = 1.00.

### 3.4. NSC-UHPFRC-Sst

**Figure 13:**
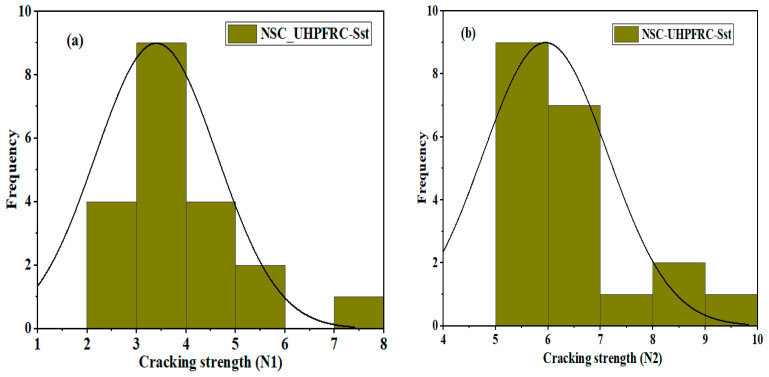
Distribution plots of NSC-UHPFRC-Sst impact test results: (**a**) *N*1 and (**b**) *N*2.

**Figure 14:**
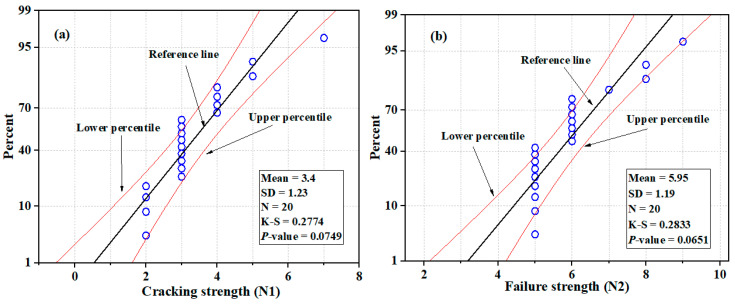
Probability plots of impact test results for NSC-UHPFRC-Sst: (**a**) *N*1 (**b**) *N*2.

The U-shaped NSC-UHPFRC specimens treated with smooth surface treatment methods showed the lowest ability to withstand repeated impact loads. The first crack occurs easily when the impact is dropped. Most of the specimens exhibited a first crack with three blows; only one specimen withstood seven blows to induce first crack strength. Upon the addition of two impact blows, the specimens undergo complete failure, as noted in Table 4. The statistical indicators of mean, SD, and COV were 4.3 blows, 1.23 blows, and 35.3% at the initial crack phase, respectively. Figure 13 and Figure 14 show the distribution and probability plots of the impact strength data of NSC-UHPFRC-Sst. The impact test at the first crack stage nearly follows a normal distribution, as depicted in Figure 13a. In contrast, the impact data at the fracture stage does not follow a normal distribution (see Figure 13b). The normal probability plots at the two cracking phases are shown in Figure 14a,b, which shows that data points are scattered around the fitting line. The Kolmogorov–Smirnov (K-S) test was used to prove the distribution results at a 0.05 significance level, and the results indicated that *N*1 had a *p*-value = 0.0749 and *N*2 had a *p*-value = 0.0651. The results are consistent with normal distribution analysis. 

### 3.5. NSC-UHPFC-Gst 

**Figure 15:**
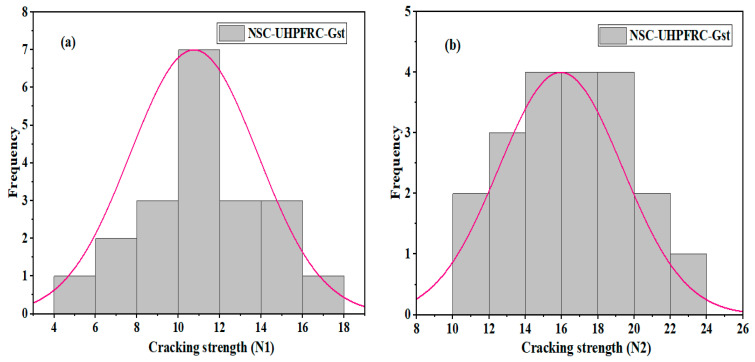
Distribution plots of NSC-UHPFRC-Gst impact test results: (**a**) *N*1 and (**b**) *N*2.

**Figure 16:**
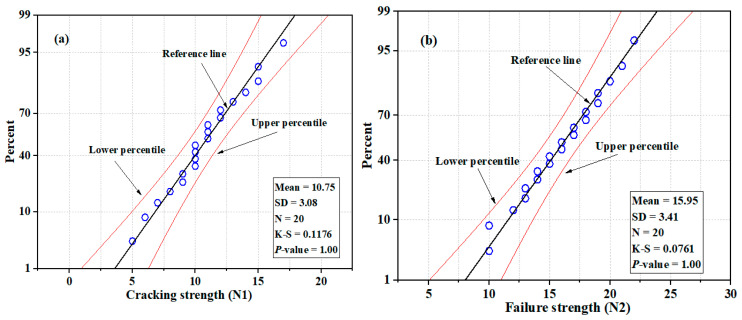
Normal probability plots of impact test results for NSC-UHPFRC-Gst: (**a**) *N*1 (**b**) *N*2.

The impact test results of U-shaped NSC-UHPFRC-Gst samples were in the range of 5 blows–15 blows at the initial crack stage and in the range of 10 blows–22 blows at the failure stage, as summarized in Table 4, with mean and standard deviation values of 10.75 blows and 3.1 blows, respectively, at the first crack stage and 15.95 blows and 3.41 blows, respectively, at the fracture stage. The *N*1 and *N*2 values in this group had a coefficient of variation of 27.89% and 20.84%, respectively. Grooving surface treatment showed better results compared to the smooth surface technique. However, natural fracture demonstrated better bonding strength at resisting impact compared with the grooving surface adopted in this study. Figure 15 and Figure 16 indicate normal distributions; probability was at a 95% confidence level for both *N*1 and *N*2, and the *p*-values of *N*1 and *N*2 for NSC-UHPFR-Gst were plotted. The plots show the impact strength data at the first stage, and the fracture stage follows a normal distribution, with distribution values located near the mean point in the overlaid curve, and many data points concentrated at the fitted line, which is nearly normal. The K-S test results for *N*1 and *N*2 at the 95% confidence level had *p*-values = 0.999.

### 3.6. Results of the Ensemble Machine Learning Models

This paper utilized the Python programming language to develop two ensemble machine-learning models. Each developed model was validated via a 10-fold cross-validation procedure [8,40]. XGBoost and CatBoost ensemble models were trained and evaluated using a dataset. The dataset included the first fracture strength (*N*1) compressive strength of composite (Cfc), Flexural load (P), and density (ρ) as the input variables. Failure strength (*N*2) was the target variable in the dataset.

#### 3.6.1. Sensitivity Analysis

**Figure 17:**
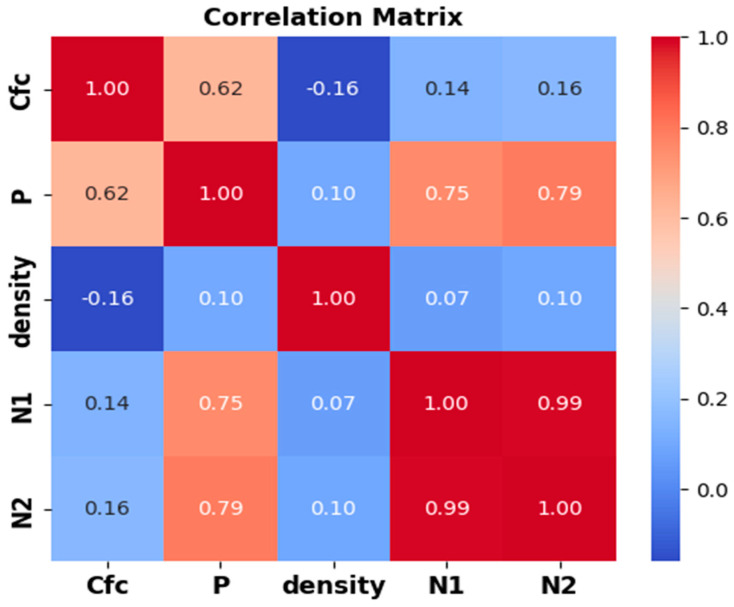
Pearson correlation matrix of the dataset.

**Figure 18:**
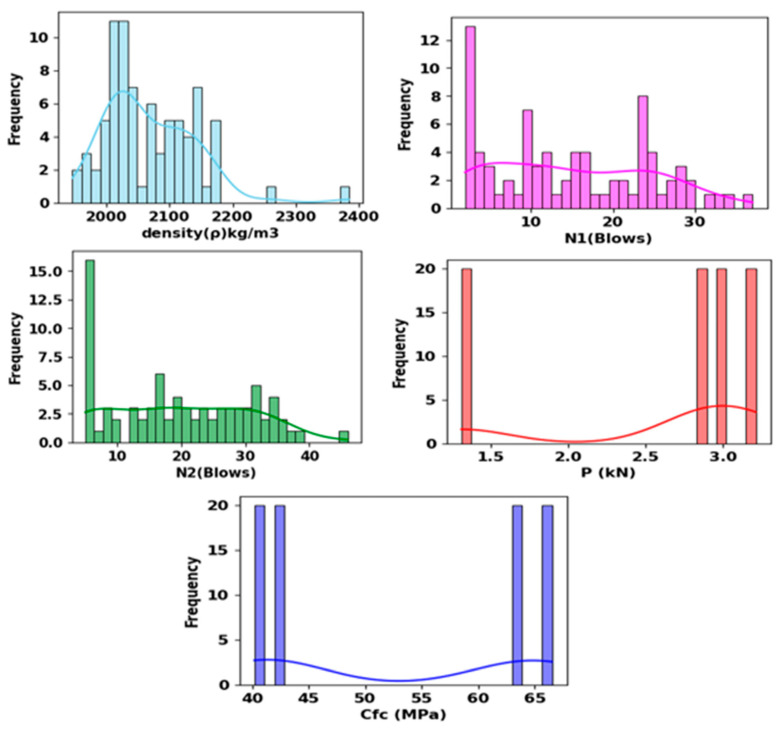
Relative distribution of the dataset parameters.

The efficacy of ML techniques depends mostly on the capability of the input variables. Numerous input parameter involvement can lead to overfitting and complicate the generated model [41]. On the other hand, more input parameters can lead to accurate models. Therefore, as illustrated in Figure 17, sensitivity analysis employing Pearson correlation was utilized in this research to investigate the most credible input features for forecasting *N*2. Table 4 summarizes the statistical indicators of the dataset. Sensitivity analysis of the dataset showed that all the input variables are positively correlated with *N*2. However, *N*1 is the most relevant variable, followed by the Flexural load, with a correlation coefficient value of 0.77. Moreover, compressive strength of the composite and density are correlated with *N*2, with correlation coefficient values of 0.32 and 0.31, respectively. The frequency distribution of the datasets is shown in Figure 18. From Figure 18, some of the variables follow the normal distribution, for example, density and *N*2, while some variables do not follow the normal distribution. 

#### 3.6.2. XGBoost Model Results

**Figure 19:**
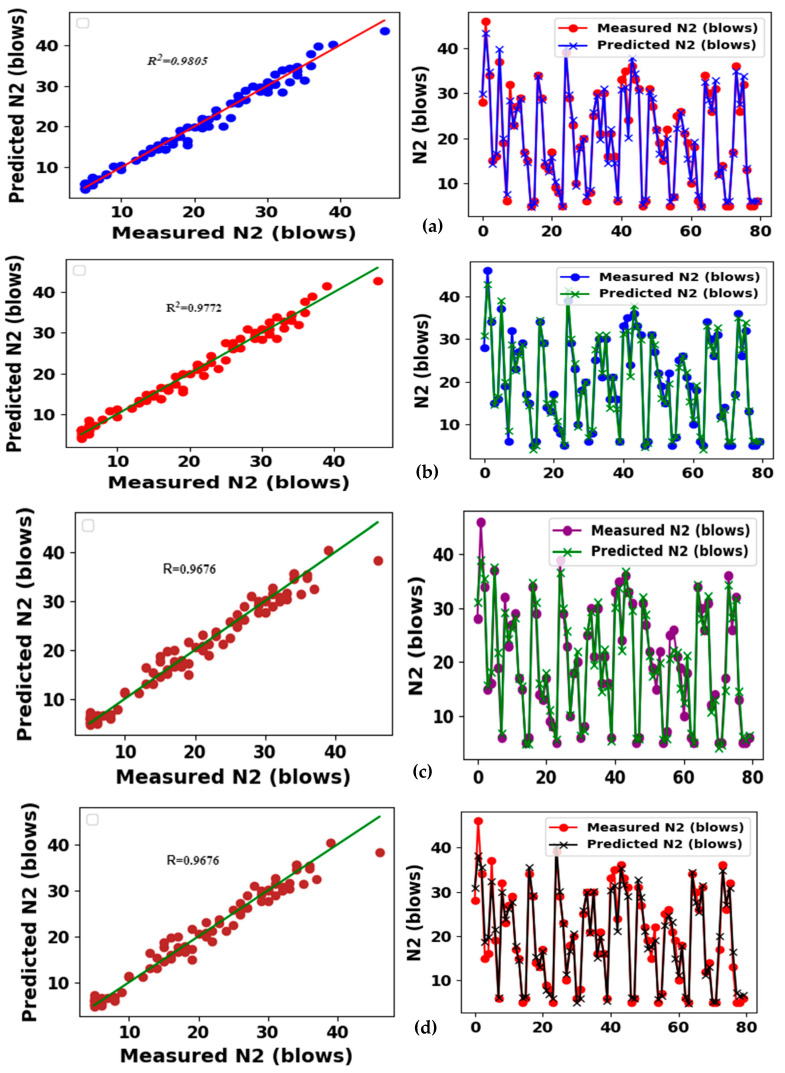
Scatter and line plots of the relationship between the predicted *N*2 and measured *N*2 values based on the (**a**) XGBoost, (**b**) Catboost (**c**), SVM and (**d**) GLM models.

The prediction skills of the XGBoost model yielded a good relationship between the forecasted *N*2 and observed *N*2 values at both the training and testing stages. The model achieved an R^2^ value of almost 0.999 at the training stage and an R^2^ value of 0.9644 at the testing phase. The model revealed a lower value of performance indicators, as presented in Table 5. The model exhibited MSE = 0.000035, RMSE = 0.0019, and MAE = 0.0014 blows at the training phase. XGBoost outperformed all other models. Additionally, high prediction accuracy was achieved with Catboost, and reasonable prediction accuracy was obtained at the testing stage, showing better performance than SVM and GLM models at the training stage. The performance of the XGBoost model is shown in Figure 19a.

#### 3.6.3. CatBoost Model Results

As obtained by the XGBoost model, CatBoost also predicts *N*2 with good accuracy. The *R*^2^ of the CatBoost model at the training phase is 0.994, which is remarkably close to that of the XGBoost model. Additionally, Catboost outperformed the other models at the testing phase, with an *R*^2^ value of 0.9671, as shown in Figure 19b, which is slightly lower than the accuracy obtained with the SVM and GLM models. MSE, RMSE, and MAE values of 0.0368, 0.2512, and 0.1950 blows were found for the CatBoost model, respectively, at the testing stage, indicating lower performance compared to XGBoost in terms of the metrics used. Furthermore, the measures of performance applied to the ensemble models had lower errors compared with the two ensemble models, as outlined in Table 5. 

#### 3.6.4. SVR and GLM

The SVM also performs very well at predicting the failure strength (*N*2), with an *R*^2^ value of 0.9772, as presented in Figure 19c. This shows a higher performance accuracy than XGBoost and CatBoost, but slightly lower performance accuracy compared to a general linear model. The MSE, RMSE, and MEA values of SVM at the testing stage were 2.1014, 1.4104, and 1.1383, respectively. Assessment of the failure strength (*N*2) by the GLM model yields the highest coefficient of determination at the testing stage, giving an *R*^2^ value of 0.9805, in contrast to XGBoost, CatBoost, and SVM as indicated in Table 5 and depicted in Figure 19d. MSE, RMSE, and MAE values of 1.88784, 1.3000, and 1.0395, respectively, were obtained at the testing phase using the GLM model, which indicates higher performance compared with CatBoost and SVM. 

**Figure 20:**
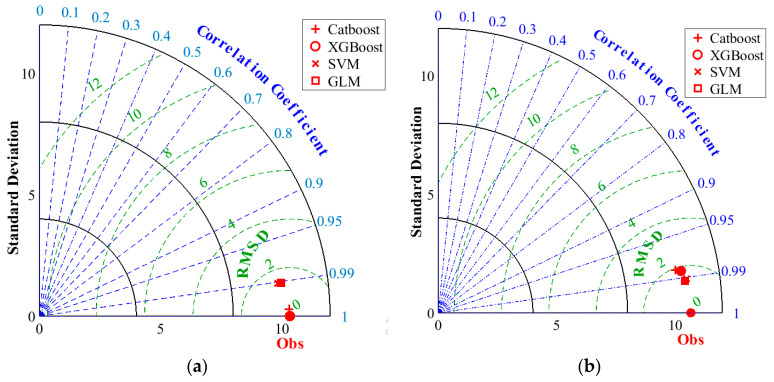
Taylor diagrams displaying model performance: (**a**) training phase and (**b**) testing phase.

The four (4) developed models, Catboost, XGboost, SVR, and GLM, were also analyzed based on the two-dimensional space diagram called the Taylor diagram (Figure 20), which graphically shows the measured and predicted values [42]. The Taylor diagram was recognized for its accurate judgment [43]. Two measurements, SD and correlation (R), were combined to build the Taylor diagram [44]. The primary purpose of this diagram is to evaluate various performance metrics in one combination and statistically compute the degree of similarity between the observed and anticipated *N*2 values. It can be noted that in both the training and testing phases (Figure 20a,b), the best performance results for the XGboost models, with an R-value = 0.999, were obtained at the training stage. Based on the data, the specified indicator represents the level of prediction accuracy for XGboost. As a result, XGboost outperformed other models because the observed locations were closer to the computed values. This is further supported by the high value of SD that was attributed to the XGboost model. In general, overestimation occurs when the SD of computed values exceeds the SD of measured values.

**Figure 21:**
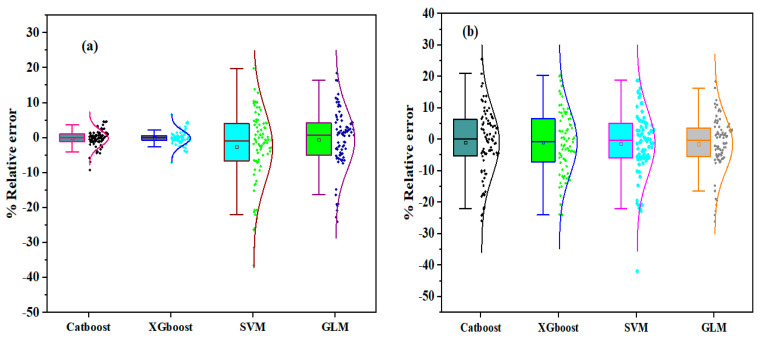
Boxplots displaying relative error distributions: (**a**) training and (**b**) testing stages.

In addition, the model performance was evaluated using the percentage relative error, shown in Figure 21. The relative error achieved by each model was compared at the two modeling stages, as depicted in Figure 21. From Figure 21a, Catboost and XGboost proved to be the best models, with the least and highest relative error distributions for forecasting *N*2. The Catboost and XGboost models’ first and third quartile (Q1 and Q3) values appeared close to one another, while GLM appeared to be the best-performing model at the testing stage.

## 4. Conclusions

The study investigated the bonding behavior of composite U-shaped NSC-UHPFRC samples using multiple drop-weight impact testing methods. The interface between the NSC substrate and the UHPFRC layer was treated with three surface treatment systems: grooving, natural fracture, and smoothing techniques. Ensemble machine learning algorithms (comprising XGBoost and CatBoost), SVM, and GLM, were employed to train and test the simulation dataset to estimate the impact strength (*N*2) of the U-shaped NSC-UHPFRC specimen. The findings of the research are summarized, and the conclusions are stated below:

(1) The impact test result indicated that surface treatment plays a significant role in ensuring sufficient bond strength at the interface of NSC-UHPFRC composites, and the bond behavior between the NSC substrate under natural fracture and the UHPFRC layer can provide sufficient bond strength at the interface, resulting in a monolithic structure that can withstand dynamic loads under repeated drop-weight impact stress.
(2) The reference NSC specimen requires a high number of drops to resist impact loads before initial crack (*N*1) occurrence, with the average number of drops equal to 24 blows compared to the NSC-UHPFRC composite samples. Remarkable reductions in impact strength properties were observed in all the composite U-shaped NSC-UHPFRC samples.
(3) The inclusion of steel fibers in the UHPFRC layer improved the composite U-shaped ductility, which transformed the composite specimens to a more ductile state and enhanced the impact strength of the NSC-UHPFRC sample. The DI values of the NSC-UHPFRC for each testing condition are less than unity. The COV of the impact data obtained in this work is lower than that found in several past studies that used the drop-weight impact testing approach ACI 544-2R.
(4) The two ensemble ML approaches correctly estimated the impact strength of the NSC-UHPFRC composite. The XGBoost ensemble model gave coefficient of determination (*R*^2^) values of approximately 0.999 and 0.964 at the training and testing stages. Similarly, GLM outperformed other models in the testing phase, with an *R*^2^ value of 0.9805. The performance matrices were proven using the Taylor diagram and Boxplots.

## Figures and Tables

**Table 1:** Mixture of the proportion of NSC and UHPFRC (kg/m^3^).

Materials	NSC	UHPFRC
OPC	420	1000
Fine aggregate	573	1200
Medium aggregate	1273	0.00
Water	185	232
Quartz powder	0.00	50.0
Water-reducing agent	0.63	200
Slag	0.00	200
Silica fumes	0.00	250
Steel Fiber (V_f_%)	0.00	1.0

**Table 2:** Technical indexes of micro steel fiber.

Properties	Length/mm	Diameter/mm	Aspect Ratio	Density/kg/m^3^	Tensile Strength/MPa
	13.0	0.2	65.0	7800	2850

**Table 3:** Impact test results of composite NSC-UHPFRC.

S/N	NSC		NSC-UHPFRC-Nst		NSC-UHPFRC-Sst		NSC-UHPFRC-Gst	
	*N*1	*N*2	*N*1	*N*2	*N*1	*N*2	*N*1	*N*2
1	24	28	22	26	3	5	10	14
2	27	34	23	30	7	9	12	19
3	12	17	10	15	4	6	5	10
4	25	33	28	32	3	5	11	19
5	23	32	20	26	5	8	10	16
6	28	34	25	30	2	5	12	17
7	26	35	23	27	3	6	11	15
8	20	25	17	22	2	5	8	13
9	15	24	12	16	3	6	6	10
10	23	29	21	26	3	5	10	15
11	37	46	33	37	4	6	17	21
12	17	25	15	19	3	5	9	13
13	18	23	16	21	3	5	7	12
14	24	30	21	27	2	5	10	16
15	29	36	24	29	3	6	13	18
16	25	31	23	29	4	6	11	17
17	34	39	28	33	3	6	15	20
18	32	36	27	31	2	5	14	18
19	29	34	25	31	4	7	15	22
20	14	21	19	23	5	8	9	14
Mean	24.10	30.60	21.60	26.50	3.40	5.95	10.75	15.95
SD	6.49	6.63	5.52	5.58	1.20	1.16	3.00	3.32
COV.	26.92	21.66	25.54	21.06	35.29	19.51	27.89	20.84

**Table 4:** Statistical indicator of the dataset.

	Parameters	Symbol	Units	Min	Max	Mean	STD	Kurtosis	Skewness
Input	Compressive strength of composite	*Cfc*	MPa	40.20	66.50	53.08	11.89	−1.99	0.017
	Flexural load	*P*	kN	1.31	3.21	2.58	0.75	−0.72	−1.06
	Density	*ρ*	kg/m	1946.1	2385.03	2068.62	73.24	3.21	1.24
	First crack strength	*N*1	blows	2	37	14.96	9.57	−1.04	0.29
Output	Failure strength	*N*2	blows	5	46	19.75	10.74	−1.03	0.15

**Table 5:** Model Performance.

Model	Training				Testing			
	MSE	RMSE	MAE	R^2^	MSE	RMSE	MAE	R^2^
XGBoost	0000035	0.0019	0.0014	1.0000	0.0000	0.0019	0.0014	0.9644
CatBoost	0.0638	0.2512	0.1950	0.9994	3.6676	1.6841	1.4128	0.9676
SVM	1.8663	1.3640	1.0410	0.9836	2.1014	1.4104	1.13836	0.9772
GLM	1.8784	1.3696	1.0395	0.9835	1.8784	1.3000	1.0395	0.9805

## Data Availability

Data are contained within the article.
